# Editorial Note: Energy use and the role of per capita income on carbon emissions in African countries

**DOI:** 10.1371/journal.pone.0335303

**Published:** 2025-10-24

**Authors:** The PLOS One Editors

The *PLOS One* Editors issue this Editorial Note to inform readers that we have concerns about aspects of the article’s [1] peer review.

Readers are advised to interpret the article with caution.
